# Structure Determination and Refinement of Paramagnetic Materials by Solid-State NMR

**DOI:** 10.1021/acsphyschemau.3c00019

**Published:** 2023-06-28

**Authors:** Jonas Koppe, Andrew J. Pell

**Affiliations:** † Centre de RMN à Très Hauts Champs de Lyon (UMR 5082 − CNRS, ENS Lyon, UCB Lyon 1), Université de Lyon, 5 Rue de la Doua, 69100 Villeurbanne, France

**Keywords:** solid-state NMR, paramagnetic, materials, structure, chemical shift

## Abstract

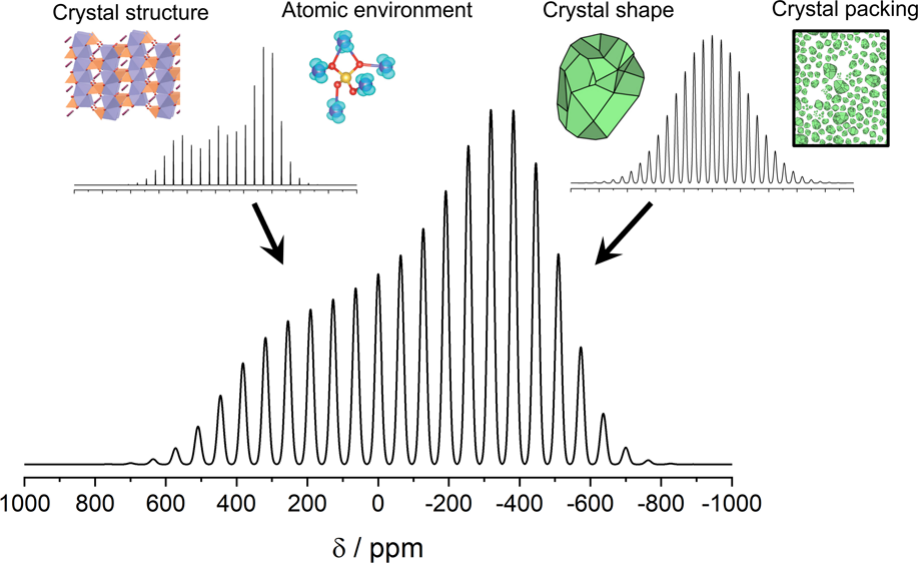

Paramagnetism in solid-state materials has long been considered an additional challenge for structural investigations by using solid-state nuclear magnetic resonance spectroscopy (ssNMR). The strong interactions between unpaired electrons and the surrounding atomic nuclei, on the one hand, are complex to describe, and on the other hand can cause fast decaying signals and extremely broad resonances. However, significant progress has been made over the recent years in developing both theoretical models to understand and calculate the frequency shifts due to paramagnetism and also more sophisticated experimental protocols for obtaining high-resolution ssNMR spectra. While the field is continuously moving forward, to date, the combination of state-of-the-art numerical and experimental techniques enables us to obtain high-quality data for a variety of systems. This involves the determination of several ssNMR parameters that represent different contributions to the frequency shift in paramagnetic solids. These contributions encode structural information on the studied material on various length scales, ranging from crystal morphologies, to the mid- and long-range order, down to the local atomic bonding environment. In this perspective, the different ssNMR parameters characteristic for paramagnetic materials are discussed with a focus on their interpretation in terms of structure. This includes a summary of studies that have explored the information content of these ssNMR parameters, mostly to complement experimental data from other methods, e.g., X-ray diffraction. The presented overview aims to demonstrate how far ssNMR has hitherto been able to determine and refine the structures of materials and to discuss where it currently falls short of its full potential. We attempt to highlight how much further ssNMR can be pushed to determine and refine structure to deliver a comprehensive structural characterization of paramagnetic materials comparable to what is to date achieved by the combined effort of electron microscopy, diffraction, and spectroscopy.

The comprehensive structural analysis of solid-state materials is typically an interdisciplinary effort.^1−3^ The determination of structural features on different length scales ranging from coarse domain morphologies to fine local atomic environments often requires the application of complementary analytical methods. Scanning and transmission electron microscopy (STEM) is ideally suited for observing domain sizes and morphological characteristics of the particles, at both their surfaces and interior. However, in particular, for more complex solid-state mixtures, obtaining high-resolution images that offer insights into the structural environment at the atomic scale might be critical. For (micro-) crystalline samples, on the other hand, unravelling molecular and/or crystal structures is routinely achieved by X-ray or neutron (powder) diffraction, which allows the determination of the average structure described by unit cell parameters, atomic distances, and space groups.^4^ Besides the general limitation to materials exhibiting long-range order (i.e., amorphous materials are not accessible), diffraction methods generally fail to provide detailed local structural information. Crucially, it is often the local structural subtleties that are key for understanding the nuanced differences in the functionalities of solid materials, the acquisition of which mostly relies on spectroscopy. This includes high-energy irradiation as for Mössbauer and X-ray absorption spectroscopy (XAS), but also low-energy microwave and radiofrequency (rf) fields for solid-state electron paramagnetic resonance (ssEPR) and solid-state nuclear magnetic resonance (ssNMR), respectively. Operating in different parts of the electromagnetic spectrum, these methods offer a variety of site-specific information that is complementary to the average structural information. For instance, XAS and Mössbauer spectroscopy potentially enable the determination of valencies, oxidation and spin states, s-electron densities, character of immediate bonds, and local coordination geometries.^5−7^ However, the provided information is spatially restricted to the immediate atomic environment, and in particular for Mössbauer spectroscopy, the application is limited to a small number of isotopes due to the scarcity of suitable radiation sources. Comparable information, e.g., electron configurations, oxidation states, charge distributions, and local atomic environments, in addition to local geometric distortions due to lattice defects or interstitial-site occupancies and compositional disorder are available via ssNMR and ssEPR spectroscopy. An overall illustration of the structural features of polycrystalline solid-state materials at different length scales available from some typically employed analytical methods is shown in Figure 1.

**Figure 1:**
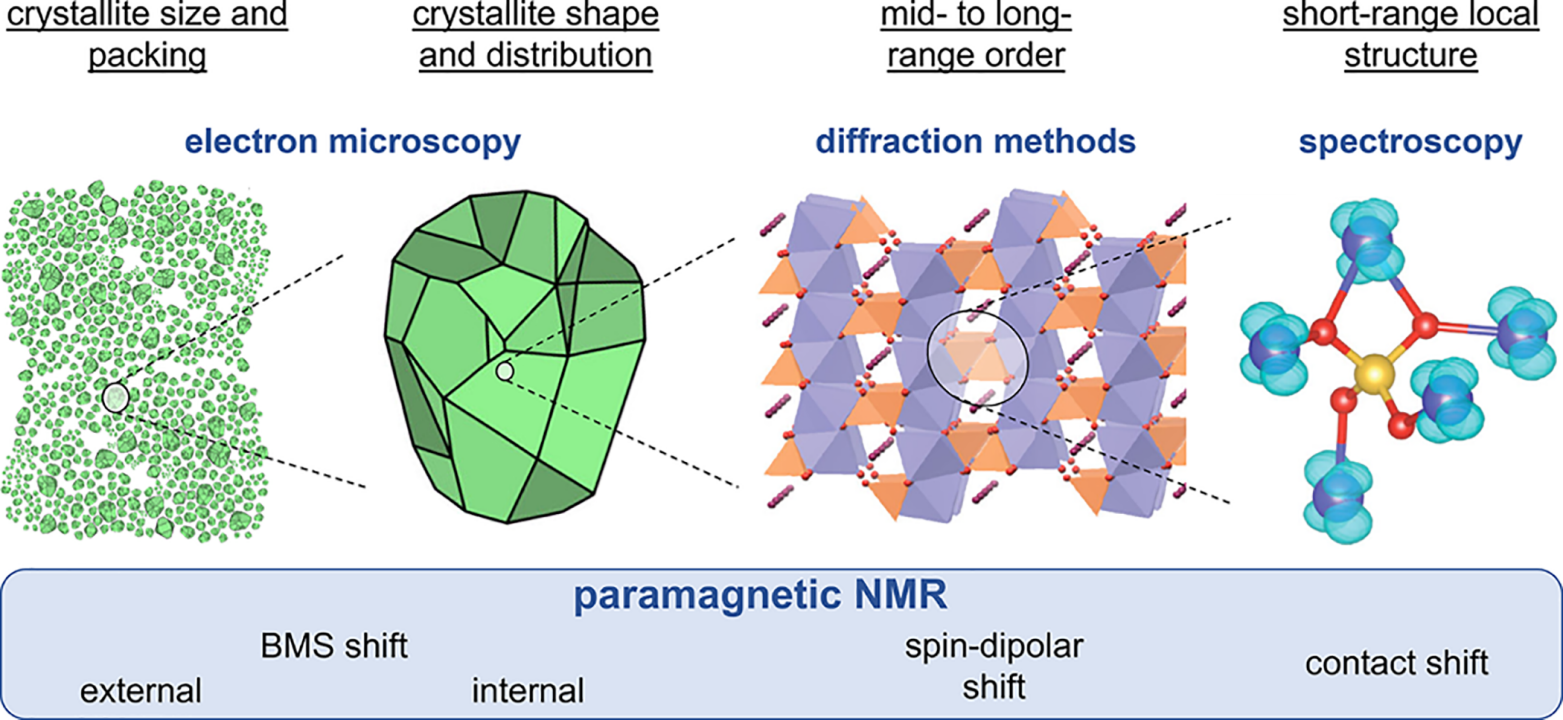
Schematic of the structural features for polycrystalline compounds at various length scales and the typically employed analytical methods used to measure them. The different contributions to the NMR shifts of paramagnetic solids that are the subject of this perspective are likewise indicated. Figures are adapted with permission from references (13) and (14). Copyright (2012) and (2019) American Chemical Society.

In particular, ssNMR spectroscopy has over the past decades enabled the study of a variety of solid-state materials, such as lipids, proteins, organic materials, polymers, metal–organic frameworks, zeolithes, glasses, and ceramics.^8^ Its broad applicability in part also stems from the large span of observable dynamic processes, including molecular rotations to chemical exchange to slow structural changes of macromolecules, and diffusion.^9^ This versatility has established ssNMR spectroscopy as a widely used analytical tool that has, however, mostly been applied to diamagnetic compounds. Nevertheless, the class of paramagnetic solid-state materials is of increasing interest in academic and industrial research, and has found several important applications, for instance, in energy conversion, storage, and transport, or solid-state lighting.^10−12^ The unique and useful macroscopic properties of solid-state paramagnetic materials are in many cases due to the unpaired electrons, that may be localized at, e.g., transition-metal (TM) ions, or delocalized over the entire material as in, e.g., metallic conductors.

While paramagnetism in principle does not degrade the quality of data obtained from STEM, XAS, and Mössbauer spectroscopy, and is a necessary prerequisite for ssEPR spectroscopy, it has several nontrivial implications for ssNMR spectroscopy. In the presence of one or more unpaired electrons, the electronic magnetic moments couple to the nuclear magnetic dipoles associated with the adjacent NMR-active nuclei via the hyperfine interaction. Since the electronic magnetic moment is several orders of magnitude larger than nuclear magnetic moments, i.e., ∼660 times larger than that of protons, the hyperfine interaction often dominates the internal spin Hamiltonian and causes a pronounced dispersion of the observed resonance frequencies, termed the paramagnetic shift, where the entire NMR signal is shifted, and paramagnetic shift anisotropy (SA), where the NMR line is broadened due to the spatial dependence of the resonance frequency. Furthermore, the hyperfine interaction typically leads to an accelerated decay of the detectable NMR signal via the paramagnetic relaxation enhancement (PRE).^11^ These characteristic phenomena on the one hand are rich in useful structural information but on the other hand pose challenges for the acquisition and interpretation of ssNMR spectra. First, the anisotropic broadening of the resonance lines over broad frequency ranges due to the paramagnetic SA results in poorly resolved spectra and also further exacerbates the inherently low sensitivity of ssNMR. The anisotropy can (in part) be averaged to enhance the signal intensity and likewise improve the resolution of the ssNMR spectrum by sample rotation, referred to as magic-angle spinning (MAS). The effect of MAS is demonstrated in Figure 2. However, for the large paramagnetic SAs observed in paramagnetic solids, meaningful improvement due to MAS requires ultrafast rotation beyond spinning rates conventionally applied to diamagnetic materials (≤ 25 kHz). Besides the anisotropic broadening caused by the paramagnetic SA, the individual resonances may additionally be separated due to the paramagnetic shift to an extent that is typically not observed for diamagnetic materials. Therefore, the overall frequency dispersion for paramagnetic solids easily exceeds several hundreds of kHz, such that conventional monochromatic rf-pulses in many cases fail to uniformly excite the full range of resonances. Further, standard NMR equipment only allows signal detection on a limited bandwidth, which might likewise be exceeded due to the paramagnetic shift and SA. Second, the fast relaxation due to the PRE severely reduces the intensity of the NMR signal, with some signals possibly being completely absent. Lastly, the paramagnetic shift as well as the paramagnetic SA both comprise several different contributions, each of with hold different structural information, as summarized in Figure 1. However, disentangling all of these individual terms that all combine in the recorded ssNMR spectrum is not straightforward.

**Figure 2:**
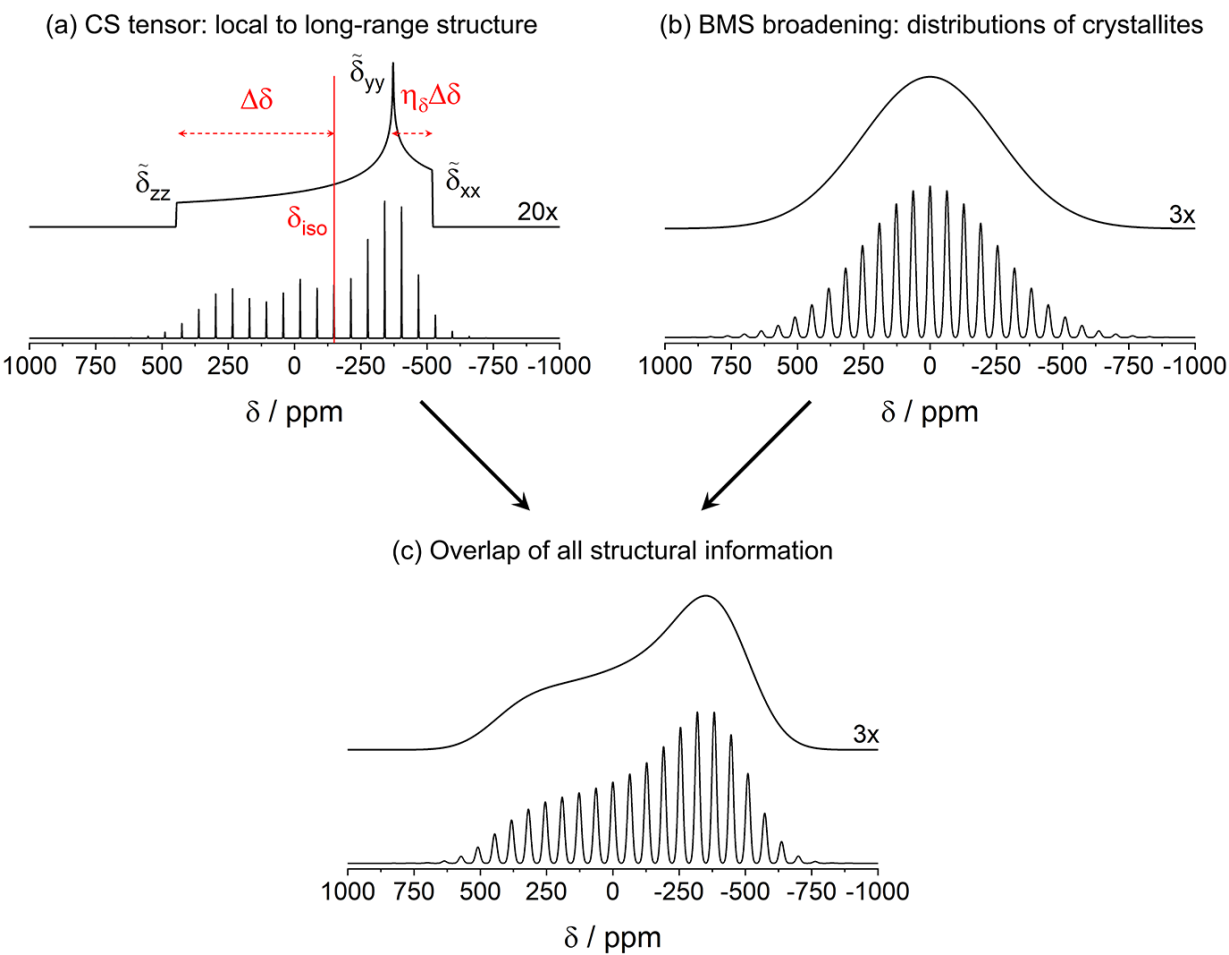
Spectral deconvolution of different types of broadenings in paramagnetic solids encoding different types of structural information. Each panel shows the simulated static and MAS (30 kHz) powder ssNMR spectra in the upper and lower trace, respectively. The static spectra have been scaled up as indicated by the numbers. (a) The typical NMR powder pattern due to a single, dominant local spin interaction that encodes local and potentially mid- to long-range structural information. A spectral interpretation of the tensor parameters introduced in eqs 2–4 is given for the static spectrum. The line shape is characterized by δ_iso_ = −150 ppm, Δδ = 600 ppm, and η_δ_ = 0.25. (b) NMR powder line shape due to a distribution of isotropic shifts and SAs, as e.g., expected from the BMS contribution in paramagnetic solids, reflecting the information about the present crystallites. The static line shape is characterized by a Gaussian distribution with an expectation value of 0 ppm, and a standard deviation of 255 ppm. Each individual spinning sideband in the MAS spectrum likewise possesses a Gaussian shape with a standard deviation of 8.5 ppm. (c) The ssNMR powder spectra in the presence of both orientational-dependent broadening and a distribution of isotropic shifts and SAs, as expected for polycrystalline paramagnetic solids.

All of the above theoretical and experimental challenges associated with the investigation of paramagnetic materials have sparked remarkable development in this field of research. On the experimental end, technological process has enabled ultrafast MAS frequencies (≥ 100 kHz). Such conditions have been proven to significantly increase the overall sensitivity, and simplify the extremely broad spectra by appropriately averaging the paramagnetic SA, and also removing nuclear dipole–dipole coupling effects.^11^ Even though ultrafast MAS uses small-diameter rotors reducing the sample volume, the corresponding small-diameter coils possess more favorable filling factors, and allow the application of very high rf-amplitudes (≥200 kHz), and thus short rf-pulses with larger bandwidths.^15,16^ Furthermore, advanced, adiabatic rf-pulse schemes and/or piece-wise acquisition of very broad NMR lines have been shown to be useful, in particular for ultrafast MAS applied to paramagnetic solids.^17−22^ In the presence of, e.g., several overlapping NMR signals or other spin interactions, two-dimensional (2D) experiments have been developed to disentangle the structural information that is not resolved in 1D spectra, even under ultrafast MAS conditions.^12,14,23−26^

Besides the progress in experimental techniques, the interpretation of high-resolution ssNMR spectra of paramagnetic materials to date is largely supported by advances in theoretical models for the chemical shift of open-shell systems. The isotropic contribution to the paramagnetic shift due to paramagnetic d-block TM ions was understood first, and described as the combination of two terms, the so-called Fermi-contact shift originating from the presence of unpaired-electron density at the center of the observed nucleus, and the pseudocontact shift (PCS) stemming from the through-space coupling between the electronic and nuclear magnetic dipoles.^27^ Subsequently, the description was extended to the full paramagnetic shift tensor, and with that to include the paramagnetic SA.^11,28−30^ The paramagnetic shift tensor was rationalized by making use of traditional EPR parameters, that are the *g*-tensor, the zero-field splitting (ZFS), and the hyperfine coupling tensor, parametrizing the atomic-level energy structure of the TM ion. This EPR formalism is often simplified by the assumption that no excited orbital states are thermally accessible, and only the ground state needs to be considered. While this leads to a valid approximation for first-row d-block TM ions in many cases, for an appropriate description of lanthanides or actinides, typically, excited states must be included, which is possible in principle but likewise more challenging.^31,32^

Equivalently, the paramagnetic shift and SA can be described based on the magnetic properties of the system, represented by an average magnetic susceptibility tensor.^33^ This approach is in particular attractive for the description of solid-state systems, as cooperative magnetism can be accounted for by adapting the form of the susceptibility tensor accordingly, and moreover, contributions to the paramagnetic shift and SA due to properties of the material as a bulk can be included easily.^11^ In addition, the magnetic susceptibility formalism allows the treatment of thermally accessible, excited states elegantly, offering straightforward application to lanthanides and actinides.^27,34−37^

Even though to date the methodologically sound theoretical and experimental techniques have enabled the acquisition of high-resolution ssNMR spectra and provide a formalism to understand the physical origin of the observed paramagnetic shifts, it remains unclear to which extent the full potential of the information hidden in these shifts can be exploited in terms of structure determination and refinement for solid-state materials. In the present perspective, this question will be addressed and furthermore pointed out where the focus of development for structure determination by paramagnetic ssNMR might be placed in the future. Therefore, a brief introduction of a theoretical framework suitable for the treatment of the NMR shift in paramagnetic materials will be presented. This will concentrate on the magnetic susceptibility formalism due to its suitability in describing paramagnetic solids. Thereafter, the information encoded in the different terms that may contribute to the overall paramagnetic shift will be described in more detail and discussed to what extent these contributions are presently included in structural investigations.

In principle, the NMR shift for a given atomic nucleus depends on the relative orientation of the (local) environment of that nucleus with respect to the external magnetic field and is accordingly represented by a 3 × 3 Cartesian tensor, known as the NMR shift tensor **δ**. The NMR shift is measured relative to a reference shift, is dimensionless, and is typically reported in parts per million (ppm). Like any Cartesian tensor, **δ** can be decomposed into the sum of an orientation-independent or isotropic part δ_iso_, and an orientation-dependent or anisotropic part **Δδ**, i.e,
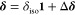
1where **1** denotes the unity tensor, and by definition **Δδ** is traceless. This decomposition has a practical meaning for the interpretation of the effect on the NMR spectrum, as the isotropic component δ_iso_ that is invariant under rotation of the system shifts the center of the corresponding resonance in the resulting NMR spectrum, while the anisotropic component **Δδ** broadens this resonance. Even though **δ** may comprise symmetric and antisymmetric components, under typical experimental conditions only the former is observed by high-field NMR. For powder samples where all possible microcrystalline orientations are observed simultaneously, the resulting NMR powder pattern in the NMR spectrum is therefore characterized by the three principal components ( with *j* ∈ *x*, *y*, *z*) of the symmetric part of **δ**. The parameters typically extracted from ssNMR spectra are demonstrated in Figure 2a: the isotropic part δ_iso_ represents the spectral position of the resonance, the anisotropy Δδ quantifies the magnitude of the broadening, and the asymmetry parameter η_δ_ indicates the deviation from the axial symmetry of the NMR shift tensor **δ**. These spectral parameters relate to the three principal components  according to
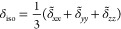
2
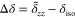
3
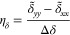
4where the  are conventionally ordered so that . As discussed in the following, in the susceptibility formalism, the shift tensor for paramagnetic materials can be expressed in terms of other measurable, tensorial quantities that relate to other physical properties of the material/TM ion. The magnetic characteristics of a paramagnetic center for instance are represented by the ensemble-average magnetic susceptibility tensor **χ**, which has the dimension [L^3^]. The fact that **χ** is a Cartesian tensor reflects that the field-induced electronic magnetic dipole moments due to the paramagnetic centers are not necessarily aligned with the external magnetic field. Their orientation and magnitude vary according to the relative orientation of the microstructure the unpaired-electron-spin density is associated with, e.g., a TM-ion complex, with respect to the external-field axis. Calculation of **χ** requires knowledge about the thermally accessible electronic orbital states, magnetic ordering, and effects of spin–orbital (SO) coupling and so includes the same information about the *g*-tensors, the ZFS, and the superexchange interactions, respectively. Analogous to the decomposition given in eq 1, the susceptibility tensor can be written as
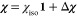
5The isotropic component χ_iso_ comprises a dominant contribution originating from the electron spin only, referred to as nonrelativistic, and a second contribution due to the SO coupling of the unpaired electron(s). The anisotropic term **Δχ** is always symmetric and is exclusively due to SO coupling, such that a system with negligible SO-coupling effects (e.g., for half-filled shells) is magnetically isotropic; i.e., the electronic magnetic dipoles are aligned with the external magnetic field, and the system is fully described by χ_iso_.

While the susceptibility tensor **χ** that represents the average magnetic properties of the paramagnetic centers encodes information about the electronic orbital structure, they do not contain information on the interactions between the electronic magnetic dipoles and the adjacent nuclear magnetic moments. For a single paramagnetic center and a given nucleus, this is described by the hyperfine coupling tensor ***C***,
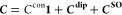
6here defined to have the dimension [L^–3^]. In this decomposition, all contributions due to SO-coupling effects are collected in the last term ***C***^**SO**^, a tensor that may comprise an isotropic, as well as an antisymmetric, and a symmetric component. Even though ***C***^**SO**^ encodes local structural information,^38^ these are to date not routinely addressed by ssNMR, and therefore are not discussed in more detail here. The first two terms on the other hand represent the nonrelativistic contribution to ***C***, which is here decomposed into the contact and the spin-dipolar contributions, denoted as *C*^con^**1** and ***C***^**dip**^, respectively. *C*^con^ corresponds to the geometric factor of the Fermi-contact coupling constant, proportional to the unpaired-electron-spin density at the center of the observed nucleus. The contact contribution is isotropic, and typically involves through-bond transfer of unpaired-electron-spin density from the paramagnetic center to the adjacent nuclei, and is thus short-range. The spin-dipolar contribution is described by the traceless and symmetric tensor ***C***^**dip**^, and represents the through-space coupling between the electronic and nuclear magnetic moment that generally acts over longer distances than the contact contribution. In the point-dipole limit, ***C***^**dip**^ corresponds to the geometric tensor analogous to that known from the dipole–dipole coupling between atomic nuclei.

As for single paramagnetic centers, a susceptibility can likewise be assigned to a specific domain within the material, e.g., a crystallite. The magnetic properties of this domain are represented by the dimensionless volume susceptibility tensor denoted as **χ**^***V***^, which can otherwise be described analogously to eq 5, i.e., as the sum of an isotropic component χ_iso_^*V*^, and an anisotropic component **Δχ**^***V***^. The magnetic dipole moment associated with a crystallite *k*, in the following denoted as **χ**^*V***,***k*^, likewise couples to surrounding atomic nuclei that may be located either within the same or within adjacent crystallites. This interaction is parametrized by the dimensionless demagnetization tensor ***N***(***r***_***k***_),^39−41^ and may be regarded as the bulk analogue to ***C***. Following classical magnetostatics, a magnetic field is induced within the crystallite due to the change in bulk magnetic susceptibility (BMS) at its boundary that opposes the external magnetic field. For the description of paramagnetic materials, where each crystallite can be considered uniformly magnetized, ***N***(***r***_***k***_) is effectively given by the surface integral of crystallite *k*, and thus encodes information about its shape. Here, ***r***_***k***_ denotes the position vector of the observed nucleus relative to crystallite *k*. In the following, the demagnetization tensor will be indicated as ***N***(***r***_***k***_) ≡ ***N***_***k***_ for brevity. Furthermore, a clear distinction must be made for ***N***_***k***_ depending on whether the observed nucleus is located inside a crystallite, in the following specified by writing ***N***_**0**_, i.e., the nucleus of interest is located in the crystallite labeled as *k* = 0, or the observed nucleus is located outside a crystallite, indicated as ***N***_***k≠0***_.^40^ In principle, ***N***_**0**_ comprises an isotropic and anisotropic component,
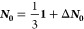
7where a nonzero Δ***N***_**0**_ indicates a deviation from a sphere of the shape of crystallite *k* = 0. On the other hand, all ***N***_***k≠0***_ are always traceless, ***N***_***k≠0***_ = Δ***N***_***k≠0***_, and encode information about the size and packing of the surrounding crystallites *k*. This might be understood from the fact that for perfectly spherical crystallites, each ***N***_***k≠0***_ is given by the through-space dipolar coupling tensor between the magnetic moment of the observed nucleus, and the magnetic dipole associated with the entire crystallite *k*.^11^ For more complex crystallite shapes, this is still a reasonable approximation for distances |***r***_***k***_| greater than the dimensions of crystallite *k*. It is emphasized that the distinction between ***N***_**0**_ and ***N***_***k≠0***_ reflects the resemblance to the hyperfine coupling tensor ***C***, and comprises an effective “contact” term due to ***N***_**0**_, and an effective “dipolar” term due to ***N***_***k≠0***_.

Generally, the NMR shift tensor **δ** for polycrystalline materials can be decomposed into contributions based on the respective spatial extent, i.e., into a contribution stemming from local spin interactions **δ**^**CS**^, often referred to as the chemical shift tensor, and a contributions due to BMS effects **δ**^**BMS**^, i.e.,
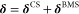
8For both of these terms, a distinction can be made between an orbital contribution also present in diamagnetic materials where the total electron spin is zero, and a open-shell contribution due to unpaired electrons. Accordingly, the chemical shift tensor **δ**^**CS**^ for paramagnetic materials can be decomposed as
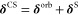
9Here, **δ**^**orb**^ denotes the orbital contribution, and even though experimentally a chemical shift based on the full **δ**^**CS**^ is measured, it may be assumed that appropriate referencing with a suitable diamagnetic analogue of the system and/or numerical calculations enable reliable extraction of **δ**^**S**^, the contribution due to unpaired electrons. **δ**^**S**^ can be expressed as the matrix product of the magnetic susceptibility tensor **χ**, and the hyperfine coupling tensor ***C***,
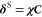
10Note that eq 10 represents the product of two tensors, one that reflects the electronic magnetic properties of the regarded domain, and a second one that represents the coupling between the electronic magnetic moment associated with this domain to the nucleus of interest. The tensor product in eq 10 comprises six cross terms that are summarized in Table I. Based on the respective components of ***C***, the following terminology might be established: the two cross terms (i) and (ii) containing the contact contribution *C*^con^ are referred to as the contact shift and contact SA, respectively. Both scale with the unpaired-electron-spin density at the center of the nucleus, i.e., delocalization of unpaired-electron-spin density onto the observed nucleus causes a positive frequency shift, while polarization may in turn lead to a negative shift. This is demonstrated for the contact shift in Figure 3a using the calculated spin-density distribution of vanadocene.^42^ The pair of cross terms (iii) and (iv) contain the element ***C***^**dip**^, and are therefore referred to as the spin-dipolar shift and spin-dipolar SA. Note however that the spin-dipolar shift is due to the isotropic component of term (iv), i.e., the cross term between ***C***^**dip**^ and the anisotropic part of the susceptibility tensor **Δχ**, and is thus due to the SO-coupling contribution to **χ**. It is commonly referred to as the pseudocontact shift (PCS) δ^PC^, and in the point-dipole approximation adopts the particularly useful and well-known expression,^43^
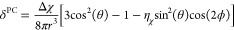
11

**Figure 3:**
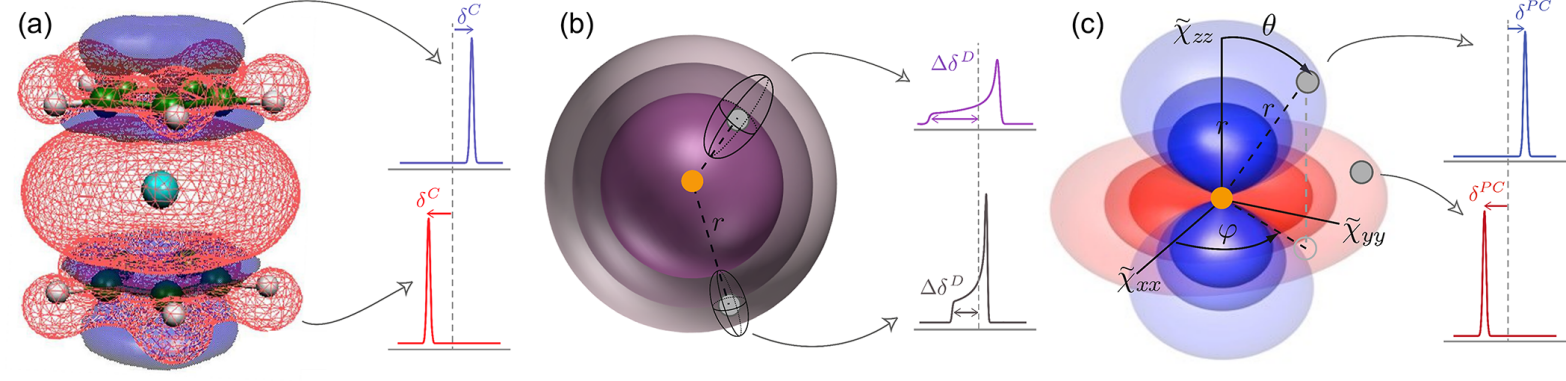
Effect of structural features on the most commonly observed paramagnetic NMR shift contributions. Each panel shows the structural element with the respective isosurface, and the expected NMR shift for: (a) the contact shift (term (i) in Table I, here indicated as δ^C^). Delocalization (red, positive shift), and polarization (blue, negative shift) of unpaired-electron-spin density onto the observed nucleus depending on its exact position is demonstrated for vanadocene, with the spin-density distribution taken from reference (42). (b) The spin-dipolar SA (term (iii) in Table I, here indicated as Δδ^D^), and (c) the PCS (isotropic component of term (iv) in Table I).^44^ For (b) and (c), the observed nucleus and the paramagnetic center are indicated as gray and golden circles, respectively. The isosurfaces show for (b) the size of the spin-dipolar SA in violet depending on the distance between the observed nucleus and the paramagnetic center, and for (c) the size and sign for the PCS in red (positive) and blue (negative) depending on the relative position of the observed nucleus in the PAS of the magnetic susceptibility tensor **χ**. Its principal values are given by , , and . The unpaired-electron-spin density in (a) is adapted from reference (42), and reproduced from Hrobárik, P. et al., The Journal of Chemical Physics, 126, 024107 (2007), with the permission of AIP Publishing. (b) and (c) are adapted from reference (44), and are reproduced from Pintacuda, G. and Kervern, G., Paramagnetic Solid-State Magic-Angle Spinning NMR Spectroscopy in Modern NMR Methodology, Springer (2012), with permission from Springer Nature.

**Table I:** Summary of the Local Terms (i–vi) and BMS Terms (a–d) Contributing to the Paramagnetic Shift^a^

Group	Term	**δ**	Rank	Type	Information
con	(i)	χ_iso_*C*^con^	0^b^	NR+SO	Precise measure for the immediate bonding geometry of the paramagnetic center
	(ii)	**Δχ***C*^con^	2	SO	
					
dip	(iii)	χ_iso_***C***^**dip**^	2	NR+SO	Spatial proximity and orientation of the nuclei with respect to the paramagnetic center.
	(iv)	**Δ**χ*C*^**dip**^	0^c^, 2	SO	
					
SO	(v)	χ_iso_***C***^**SO**^	0,2	SO	
	(vi)	**Δ**χ*C*^**SO**^	0,2	SO	
					
BMS,in	(a)	–χ_iso_^*V*,0^Δ***N***_0_^*T*^	2	IBMS	Shape and distribution of shapes of the crystallites. Isotropic part of (b) dominates the isotropic BMS shift.
	(b)	–Δ**χ**^***V***,**0**^Δ***N***_**0**_^*T*^	0,2	ABMS	
					
BMS,ext^d^	(c)	–χ_iso_^*V*,k^Δ***N***_**k**_^*T*^	2	IBMS	Size and packing of the crystallites. (c) presumably dominates the distribution of paramagnetic SAs.
	(d)	–Δ**χ**^***V***,**k**^Δ***N***_**k**_^*T*^	0,2	ABMS	

a The ranks represent the effect on the NMR line; zero-rank terms result in an isotropic shift, while second-rank terms cause SA and resonance broadening. Potential first-rank terms are omitted as they are not observed under high magnetic fields. The type of each term reflects whether they stem from non-relativistic (NR) or spin-orbital (SO)-coupling effects for the local contributions, or from isotropic (I) or anisotropic (A) BMS effects for the BMS contribution.

b Contact shift. NMR parameter most commonly considered for paramagnetic inorganic materials.

c Pseudocontact shift. Typically used for proteins.

d The summation over all crystallites *k* ≠ 0 in the term symbols has been omitted for clarity.

Then, the PCS depends on the anisotropy and asymmetry of the susceptibility tensor **χ** (cf. eqs 3 and 4), and on the spherical coordinates (*r*, θ, ϕ), representing the relative position of the nucleus in the principal-axis frame of **χ**. The spin-dipolar SA comprises contributions from both cross terms (iii) and (iv) involving ***C***^**dip**^, where **χ**_iso_***C***^**dip**^ has a dominant nonrelativistic contribution. The geometrical information encoded in the spin-dipolar SA and the PCS, and how they influence the ssNMR spectrum in the point-dipole limit are depicted in Figure 3b and c.^44^ For the former shown in (b), only the distance *r* between the observed nucleus (gray circle) and the paramagnetic center (golden circle) determines the size of the SA (denoted as Δδ^D^ in Figure 3b). This is represented by the spherical isosurfaces for Δδ^D^. Since all orientations of *r* with respect to the external magnetic field are present in a powdered sample, the typical inhomogeneous powder lineshapes are obtained. On the other hand, for the PCS the distance *r* and the relative position (*r*, θ, ϕ) of the nucleus in PAS of the susceptibility tensor **χ** (the principal values are denoted as , , and ) determine the resulting isotropic resonance shift, as indicated in Figure 3c. Note that the isosurfaces shown in Figure 3b and c refer to isolated paramagnetic centers. However, in a crystalline lattice of a paramagnetic solid, these isosurfaces become more complicated, as they are given by the sum from an ensemble of TM ions.

It should be emphasized that a drastic simplification can be made for magnetically isotropic paramagnetic centers, as e.g., the case for TM ions with half-filled shells in high-spin complexes. Then all SO-coupling effects can be disregarded, such that the paramagnetic shift and SA are exclusively due to the nonrelativistic parts, i.e., only terms (i) and (iii) in Table I,
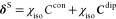
12

These are referred to as spin-only systems, as summarized in Figure 4. It is emphasized that eq 12 might be seen as the paramagnetic analogue to the magnetic dipole coupling between nuclear spins in diamagnetic materials. This is described by the isotropic through-bond *J*-coupling and anisotropic through-space direct dipole–dipole coupling.

**Figure 4:**
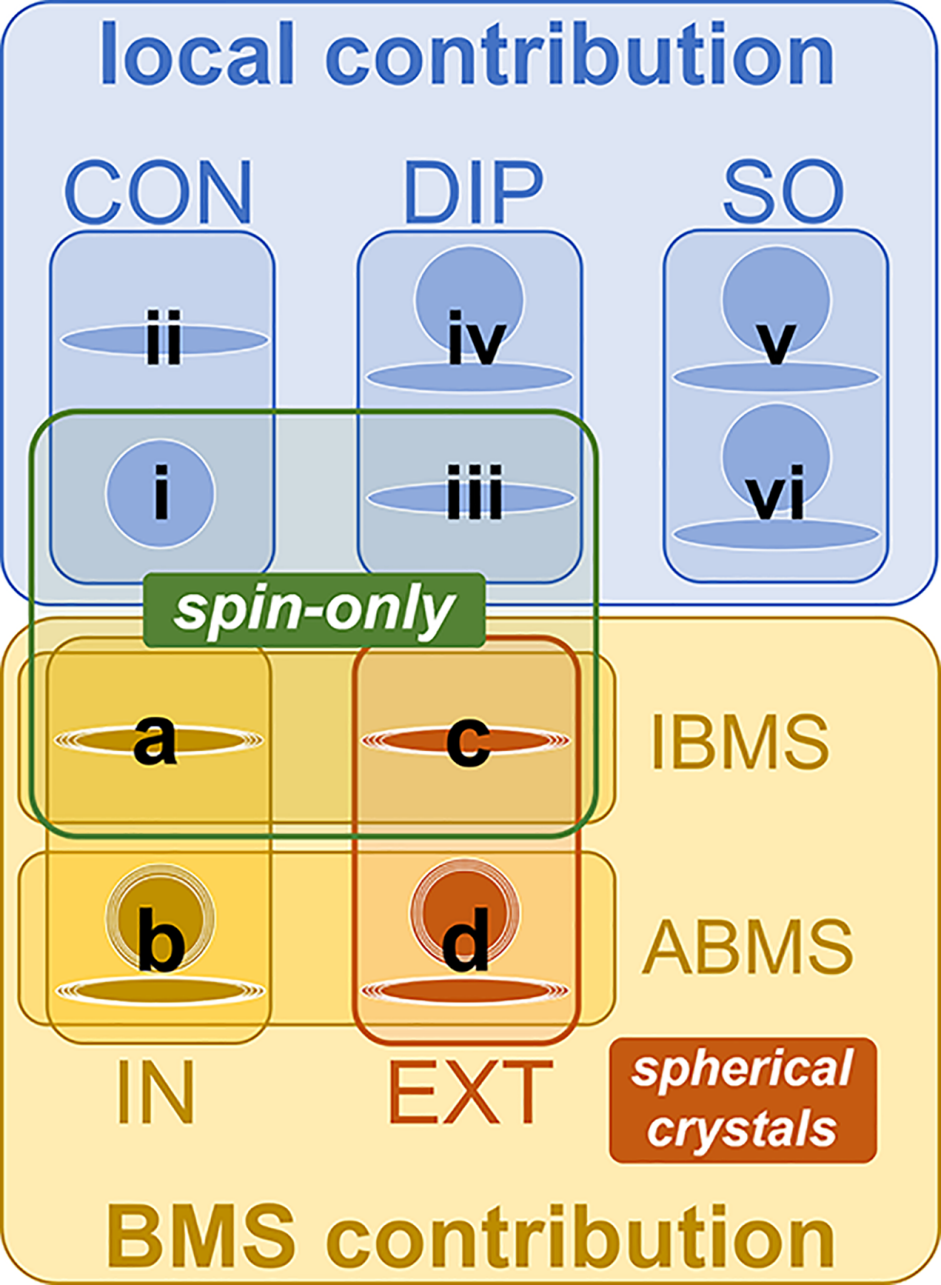
Relationship between the different terms from Table I. The spatial characteristics of the present terms are symbolized by circles for the isotropic (zero-rank), and ellipsoids for anisotropic (second-rank) components, or both. For the BMS terms, the expected distributions of contributions are indicated by the less clearly defined geometrical figures. The relative sizes of the different shapes and distributions do not represent the respective magnitudes. The simplifications for the two particular cases of nonrelativistic spin-only systems and spherical crystals are likewise considered.

In addition to the chemical shift contribution **δ**^**CS**^, the NMR shift tensor **δ** given in eq 8 comprises a second term due to the magnetic properties of the microcrystalline domains of the sample, commonly termed BMS effects, which captures the interaction between the observed nucleus and a whole crystallite. While for diamagnetic materials this term might comprise the resolution of ssNMR spectra, however to a minor extent,^39^ for paramagnetic polycrystalline solids the corresponding BMS shift tensor **δ**^**BMS**^ contributes significantly to NMR shift tensor **δ**, and can be expressed as
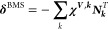
13where the summation includes all crystallites *k* of the sample, and the superscript *T* denotes the transpose matrix. Note that analogous to the paramagnetic contribution **δ**^**S**^ to the chemical shift tensor, this is the matrix product of the volume susceptibility tensor that represents the magnetic properties of a crystallite *k*, **χ**^*V***,***k*^, and the demagnetisation tensor ***N***_***k***_, reflecting the coupling between the magnetic moment associated with the crystallite *k* to the nucleus of interest.^39−41^ The full BMS-shift tensor may be expanded in two parts,
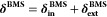
14

The first terms **δ_in_^BMS^** represent the paramagnetic shift and SA due to the interactions of the observed nucleus with the crystallite it is located in, and is thus given by the cross term of the BMS tensor of crystallite *k* = 0, **χ**^***V***,**0**^, and its demagnetization tensor ***N***_**0**_.^40,45^ In principle, this yields four cross terms, since both **χ**^***V***,**0**^ and ***N***_**0**_ comprise an isotropic and anisotropic components each (cf. eqs 5 and 7). However, all paramagnetic centers in close proximity to the observed nucleus, located in the so-called Ewald sphere around it, give rise to the local contribution captured in the term **δ**^**S**^. The Ewald sphere is assumed to be much smaller than the crystallite *k* = 0 within which the observed nucleus is located, and to avoid double counting, all BMS effects involving paramagnetic centers within the Ewald sphere must be excluded from **δ_in_^BMS^**. Effectively, this cancels out the two cross terms containing the isotropic part of ***N***_**0**_, and only the two cross terms comprising Δ***N***_**0**_ remain (terms (a) and (b) in Table I). The second BMS contribution **δ_ext_^BMS^** is due to the coupling between the magnetic moment of the observed nucleus and the electronic magnetic dipoles associated with all other crystallites *k* ≠ 0 that surround crystallite *k* = 0, i.e., the sum over all products of the respective BMS tensors **χ**^***V*****,*****k*****≠0**^ and demagnetization tensors ***N***_***k≠0***_. As the latter are always traceless, this results in the sum of two cross terms, denoted as (c) and (d) in Table I. Note that in principle, a third BMS contribution could be additionally considered, originating from the entire sample packed into the sample container. The container itself can be assumed uniformly magnetized with an associated magnetic moment that may couple to the magnetic dipole of the observed nucleus.^46^ This contribution varies with the overall shape of the sample container and can be treated analogously to **δ_in_^BMS^**, but it is not further discussed here.

The four BMS terms (a)–(d) in Table I are often regrouped according to whether they contain the isotropic or anisotropic component of the respective susceptibility tensor, χ_iso_^*V*^ or **Δχ**^***V***^, commonly referred to as isotropic and anisotropic BMS (IBMS and ABMS) parts, respectively.^47^ For spin-only systems, only IBMS terms (a) and (c) remain, as illustrated in Figure 4. In the special case of spherical crystallites, the description of the full BMS shift simplifies considerably, as Δ***N***_**0**_ = 0, the contribution due to **δ_in_^BMS^** vanishes, and only **δ_ext_^BMS^** needs to be considered (see Figure 4). On the other hand, as described above, the coupling between the magnetic dipole of the observed nucleus to the magnetic moments of the surrounding crystallites can be described as the coupling between two point dipoles, i.e., **Δ*****N***_***k*****≠0**_ can then be described by the geometrical part of a dipole–dipole coupling tensor.^48^

It is important to highlight that in contrast to the paramagnetic contribution to the chemical shift tensor, where **δ**^**S**^ represents the entire ensemble of observed nuclear spins, **δ**^**BMS**^ changes throughout the ensemble due to the variation of crystallite shapes, sizes, relative orientations, and packing within the sample. Therefore, the BMS contribution causes a distribution of isotropic and anisotropic resonance shifts, as likewise indicated in Figure 4. Note that the distribution of the isotropic paramagnetic shifts stems from (b) and (d), since only the ABMS terms comprise isotropic components. While both of these terms may equally contribute to the distribution of isotropic shifts, for (d) the large number of different crystallite configuration allows the application of the central limit theorem, predicting the expectation value for term (d) to be zero,^13^ i.e., there is no net shift of the overall resonance. The distribution of paramagnetic SAs in principle may arise from all four terms (a)–(d). Generally, the terms due to **δ_ext_^BMS^**, i.e., (c) and (d), are assumed to be more dominant, where again term (d) vanishes in nonrelativistic systems (spin-only, cf. Figure 4), such that the overall contribution of term (c) is presumably larger.

Concluding the discussion of terms contributing to the NMR shift due to paramagnetism, we have described that the contact contribution, stemming from the through-bond transfer of unpaired-electron-spin density, contains structural information about the immediate bonding geometries of the paramagnetic center. On the other hand, the spin-dipolar contribution, arising from the though-space coupling between the electronic magnetic moments and the nuclear magnetic dipoles, acts over larger distances and encodes medium- to long-range structure information. In addition to these terms, the BMS contribution to the NMR shift originates from the interaction of the observed nuclei with electronic magnetic dipoles associated with entire crystallites. Clearly, these dipole moments are significantly larger than those associated with single paramagnetic centers and thus couple to nuclei on much larger distances. Therefore, the BMS contributions include information concerning the shapes, sizes, and packing of the crystallites.

While in principle each of the ten different terms listed in Table I may comprise structural information on the material, not all of these terms are of practical relevance in modern ssNMR to date. In the majority of structural studies on paramagnetic solid-state materials where paramagnetic shifts and SAs are included, XRD data form the basis of the analysis, and serve as a platform for further structural refinements. In this context, data obtained from ssNMR is often used to either verify, or provide additional information not available from XRD, such as insights into the local structure and compositional disorder. In particular for microcrystalline inorganic materials, structural information has been obtained from ssNMR data via the paramagnetic contribution to the chemical shift, **δ**^**S**^, while the BMS contribution **δ**^**BMS**^ is rather viewed as an unwanted additional source of inhomogeneous line broadening, primarily reducing the spectral resolution, as e.g., in early studies of rare-earth pyrochlores, and deuterated acetylacetonate and amino-acid complexes.^49−52^ The nature of the system under investigation often allows for additional simplification; i.e., many of the shift contributions can be neglected. Studies of paramagnetic first-row TM-ion materials that have found applications as electrode materials in lithium- and sodium-ion batteries have been very successful in rationalizing the obtained ssNMR data based on the nonrelativistic terms in **δ**^**S**^.^14,26,53−61^ As described in eq 12, then the contact shift χ_iso_*C*^con^, term (i) in Table I, is the only contribution to the isotropic paramagnetic shift and is often assumed to likewise dominate the overall NMR shift. Despite the orbital shift and the PCS being present,^62,63^ these terms are often insignificant compared to the contact shift, such that an interpretation of the observed resonance positions based on the unpaired-electron-spin density at the respective nuclear site is reasonable. Its sign and magnitude have been found to be extremely sensitive toward the local structure and bonding environments, and the contact shift to date is well-established as a sensitive measure for detecting compositional or local structural disorder or defects, i.e., local effects that are usually not captured by other methods. A compelling example is the seminal study of the lithium-layered TM-ion oxides LiTMO_2_ with TM = Co, Cr, Mn, Fe, Ni, and the mixed TM-ion complexes LiTM_1/8_Co_7/8_O_2_ with TM = Cr, Ni via ^6,7^Li NMR shifts.^53,56^ These materials contain alternating layers of edge-sharing LiO_6_ and TMO_6_ octahedra such that each Li-site configuration comprises six 90° and six 180° Li–O–TM bonding motifs. Overlap between the Li 2s-orbital and the TM 3d-orbitals occurs via the different bridging O 2p-orbitals. This results either in an increase of positive unpaired-electron-spin density at the Li-site via delocalization into the Li 2s-orbital, or negative unpaired-electron-spin density at the Li-site due to polarization of the 2s-orbital.^53^ The contact shift was shown to be given by the sum of all of these so-called pathway contributions due to the surrounding TM ions in the different Li–O–TM bonding geometries, and can be used as a fingerprint to identify a specific Li-site configuration.^53,56^ The solid-state density functional theory (DFT) calculations of the individual spin-transfer-pathway contributions for the Li-sites in the mixed-TM-ion complexes LiCr_1/8_Co_7/8_O_2_ and LiNi_1/8_Co_7/8_O_2_ revealed that a Jahn–Teller distortion is expected at the Ni-site, which ssNMR data indicated is dynamic rather than static.^56^

The contact shift has also been used successfully to probe the local P environment and refine the structures suggested by XRD for the olivine-type lithium TM phosphates LiTMPO_4_ with TM = Mn, Fe, and Co,^56^ and the mixed phases LiFe_*x*_Mn_1–*x*_PO_4_ and LiFe_*x*_Co_1–*x*_PO_4_ with *x* = 0, 0.25, 0.5, 0.75, 1.^14,58^ This is demonstrated in Figure 5: in (a), the general structure expected from XRD for LiFe_*x*_Mn_1–*x*_PO_4_ is shown. The ^31^P MAS NMR spectra of the pure phases LiMnPO_4_ and LiFePO_4_ are given in Figure 5b, and accordingly represent the combined spin-transfer-pathway contributions for the all Mn–O–P-site and the all Fe–O–P-site configurations, respectively. The insets show the local structure of the P atom, which is defined by the Mn/Fe ion occupancy of the five nearest-neighbor TM sites to the PO_4_ motif, and the isotropic projections from the corresponding 2D adiabatic magic-angle turning (aMAT^14^) NMR spectra. The occurrence of a single resonance in these projections reflects that the pure phases both have the same local structure, with all five TM sites occupied either wholly by Mn or Fe ions, respectively. For the mixed phases, however, the ^31^P MAS NMR spectra are more complicated, giving rise to a superposition of several ^31^P resonances, expected for the 32 possible Fe/Mn–O–P-site configurations due to the mixed Mn/Fe occupancy of the five TM sites. This is likewise reflected by the isotropic projections from the 2D aMAT spectra, as demonstrated for LiFe_0.5_Mn_0.5_PO_4_ in Figure 5c (black line). The individual contact shifts for all P-site configurations calculated by solid-state DFT were used as fingerprints to model the experimental data (red line in Figure 5c), and each configuration was assigned to a ^31^P signal. This ultimately allowed a refinement of the structures for the mixed phases at the different stoichiometries in terms of the Fe^2+^/Mn^2+^, and later, to the Fe^2+^/Co^2+^ distribution. In addition to that, the DFT analysis of the distinct contact shifts further enabled monitoring of the changes in the TM–O–P-bond distances and angles for the different TM–O–P-site configurations within the mixed phases, which reflect even minor distortions of the TM environment occurring upon TM substitution, beyond the insights available from XRD.^14,58^

**Figure 5:**
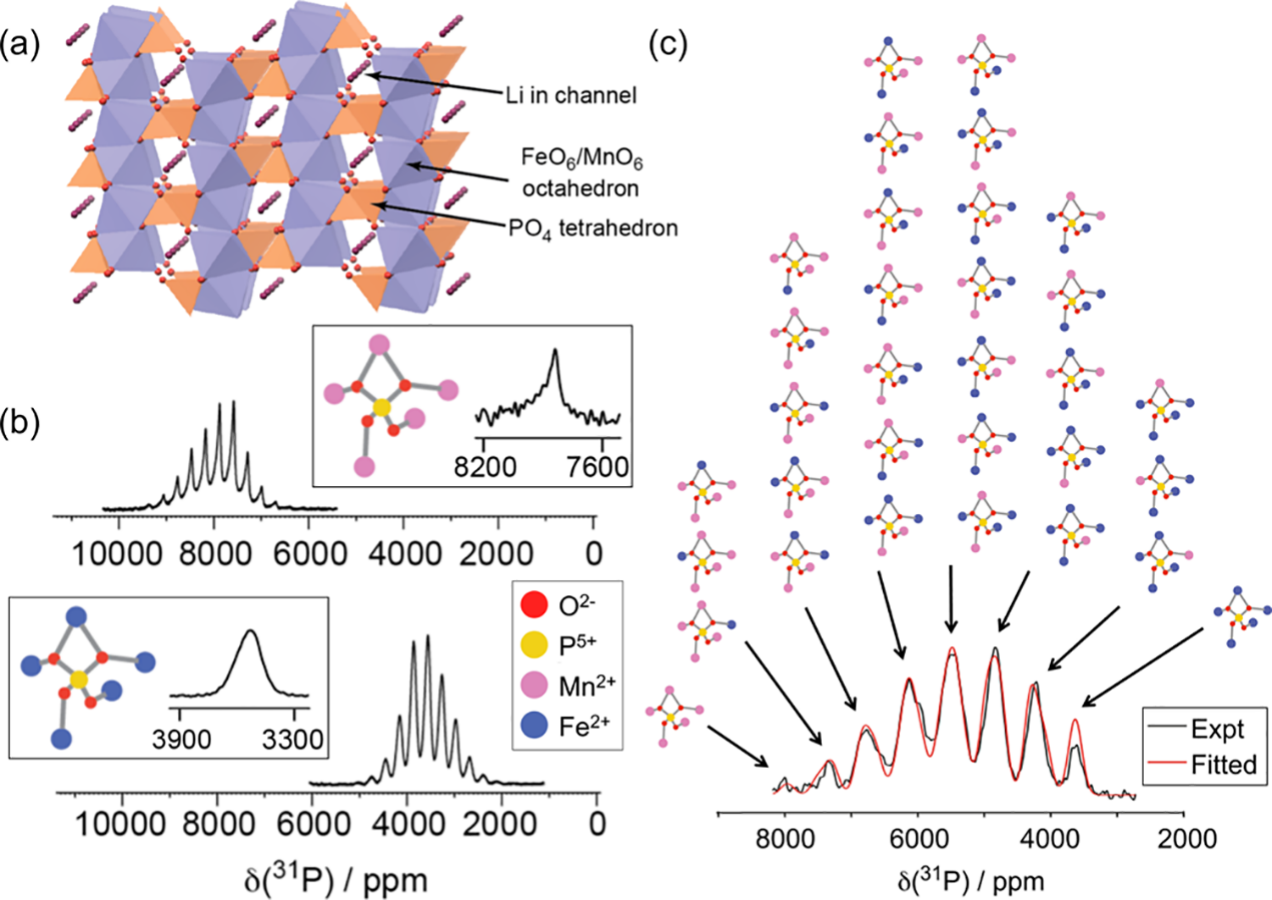
Probing compositional disorder in olivine-type lithium TM phosphates LiTMPO_4_. (a) General structure from XRD with TM = Mn/Fe. (b) ^31^P MAS NMR spectra (60 kHz MAS) for the pure phases LiMnPO_4_ and LiFePO_4_ in the top and lower panel, respectively. The insets show the structure elements and the isotropic projections from the corresponding 2D aMAT NMR spectra. (c) Isotropic projection from the ^31^P 2D aMAT NMR spectrum of the mixed-phase LiFe_0.5_Mn_0.5_PO_4_ (black line) and the numerical model (red line). Based on the respectively calculated contact shifts, each of the 32 possible P-site configurations could be assigned to the different ^31^P isotropic signals, as indicated above the spectrum. Adapted with permission from reference (14). Copyright (2012) American Chemical Society.

The same strategy was employed to investigate the local ^7^Li environment and determine the cation distribution in the mixed phases LiTi_*x*_Mn_2–*x*_O_4_ with 0.2 ≤ *x* ≤ 1.5.^61^ For lower Ti doping levels of 0.2 ≤ *x* ≤ 0.8, it is expected for the cations Li^+^, Ti^4+^, and Mn^3+/4+^ to be present, with fast electronic conduction (compared to the NMR-time scale) between Mn^3+^ and Mn^4+^. The proposed spinel-type AB_2_O_4_ average structure is shown in Figure 6a, where it is expected that Li^+^ and Ti^4+^/Mn^3+/4+^ will occupy the 8*a* tetrahedral and 16*d* octrahedral sites, respectively. A DFT calculation assuming a random distribution of Ti^4+^/Mn^3+/4+^ with Li^+^ on the tetrahedral sites is inconsistent with the experimental ^7^Li ssNMR data, as demonstrated in Figure 6b for LiTi_0.2_Mn_1.8_O_4_ (dashed red and black lines, respectively). Here, the ^7^Li MAS spectrum was modeled by combining specific local Li-site configurations, defined by the Ti/Mn occupancy of the 12 nearest TM (octahedral) sites, as likewise indicated in Figure 6b. For higher Ti doping levels, *x* ≥ 1.0, the more ordered cubic spinel shown in Figure 6c is expected to form with the cations Li^+^, Mn^2+^, and Ti^4+^ to be present. Here, a random distribution of the cations on the different tetrahedral and octrahedral sites was found to be in clear contraction with the experimental NMR spectrum. A reasonable structural model was eventually obtained by including a reverse Monte Carlo approach: starting from a distinct cation distribution, e.g., all Li^+^ on tetrahedral sites and all Mn^2+^/Ti^4+^ on octrahedral sites, cations were randomly swapped. The swaps were then accepted based on the new ^7^Li shifts. The best model, corresponding to the structure (Li_0.6_Ti_0.1_Mn_0.3_)_8*c*_[(Li_0.1_Ti_1.4_)_12*d*_(Li_0.3_Mn_0.2_)_4*d*_]O_4_, i.e., a mixture of all three cations on both tetrahedral and octahedral sites, is shown in Figure 6d for LiTi_1.5_Mn_0.5_O_4_, where the individual ^7^Li signals and corresponding structural elements are also indicated. Ultimately, this has led to a comprehensive characterization of the different stoichiometries 0.2 ≤ *x* ≤ 1.5, regarding the Li^+^, Mn^2+/3+/4+^, and Ti^4+^ cation distribution.^61^

**Figure 6:**
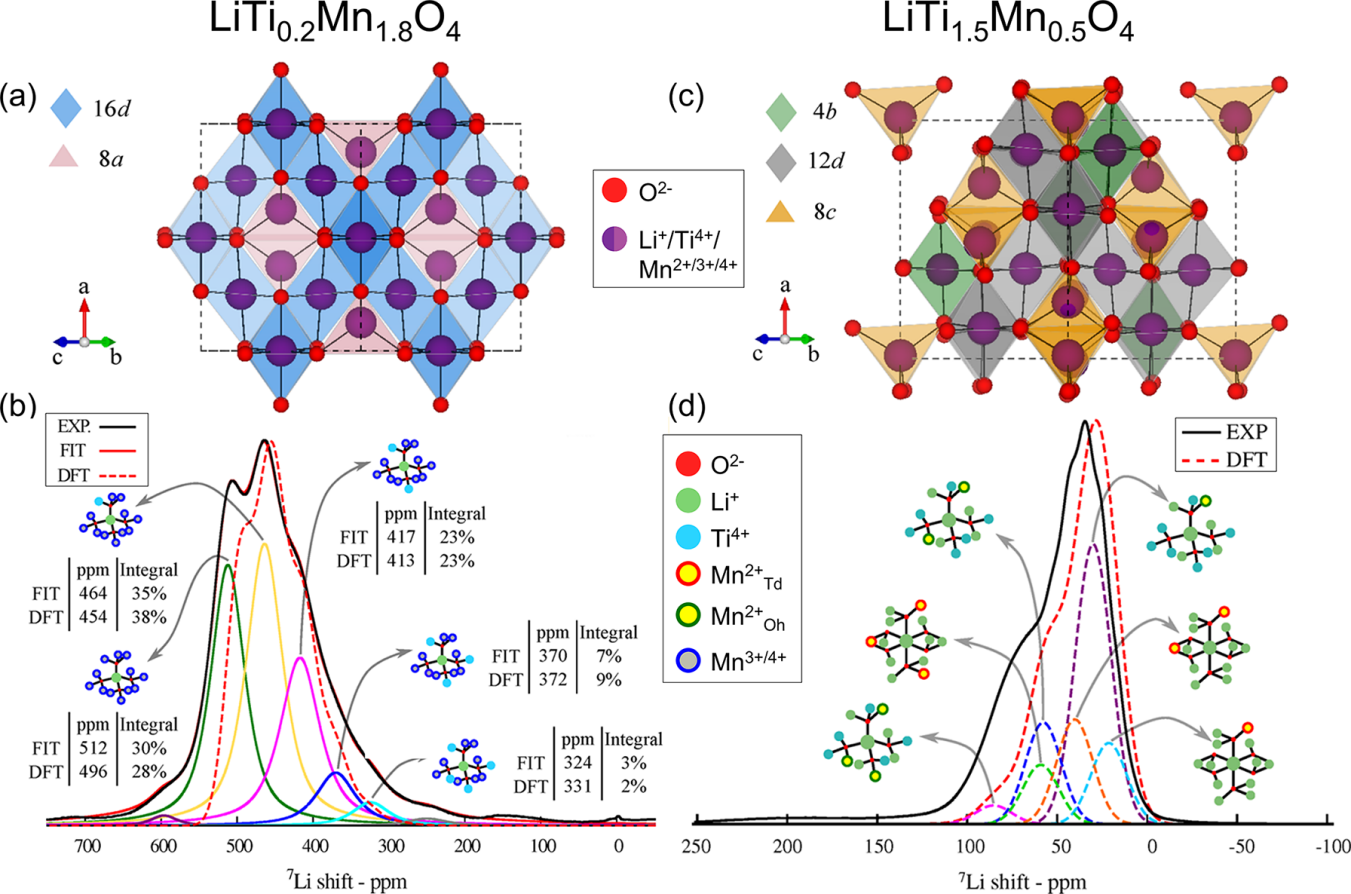
Solving the cation distribution for LiTi_*x*_Mn_2–*x*_O_4_ with 0.2 ≤ *x* ≤ 1.5. Polyhedral representation of the spinel-type AB_2_O_4_ structures in the Fd3m and the *P*4_3_32 space groups in (a) and (c), proposed for LiTi_*x*_Mn_2–*x*_O_4_ with lower Ti doping 0.2 ≤ *x* ≤ 0.8, and higher Ti doping *x* > 1.0, respectively. ^7^Li MAS NMR spectra (60 kHz MAS, black lines) of (b) LiTi_0.2_Mn_1.8_O_4_ and (d) LiTi_1.5_Mn_0.5_O_4_. The individual ^7^Li signals and corresponding structural elements are likewise indicated. In (b), the fit (solid red line) is obtained from combining the signals of the individual Li configurations (colored lines). The relative contributions of each configuration is additionally given. The spectrum computed by DFT assuming a random distribution of cations is shown by the dashed red line. In (d), the dashed red line represents the best spectrum obtained employing an inverse Monte Carlo approach, corresponding to the structural decomposition (Li_0.6_Ti_0.1_Mn_0.3_)_8*c*_[(Li_0.1_Ti_1.4_)_12*d*_(Li_0.3_Mn_0.2_)_4*d*_]O_4_. Adapted with permission from reference (61). Copyright (2018) American Chemical Society.

Computing fingerprint contact shifts to probe the local environment has also been successfully applied to paramagnetic Fe^3+^ phosphates including polymorphs of Li_3_Fe_2_(PO_4_)_3_, where local ^7^Li and ^31^P bonding geometries deviate from 90° and 180°,^55^ and to more challenging nuclear sites, e.g., the local ^17^O environment in Li_2_MnO_3_,^59^ and the local ^25^Mg environment in magnesium TM oxides.^60^

In contrast to the contact shift, the contact SA **Δχ***C*^con^, term (ii) in Table I, has rarely been employed for obtaining atomic-level structural insights in ssNMR. In principle, both terms (i) and (ii) encode information about the unpaired-electron-spin density at the respective nuclear site and might thus be employed to support the respective insights. Nevertheless, for the **Δχ***C*^con^ term, this information is encoded in the NMR powder line shape via the anisotropy of the susceptibility tensor Δχ, and is often more difficult to determine experimentally than the contact shift. While assuming the contact shift to be the dominant contribution to the overall NMR shift is often a reasonable approximation, the contact SA on the other hand is exclusively due to the effect of SO-coupling, which is typically dominated by the much larger, nonrelativistic spin-dipolar SA, or other spin-interaction anistropies, e.g., the quadrupolar broadening. A rare example where the contact SA has been shown to be significant are again the lithium TM phosphate phases LiMnPO_4_ and LiFePO_4_. Comparing the ^31^P NMR spectra shown in Figure 5b, clearly the sign of the overall anisotropy Δδ (cf. eq 3) changes from negative for LiMnPO_4_, to positive for LiFePO_4_. As it was reported that structural changes upon TM-ion substitution (Mn–Fe) are minor,^14^ the change cannot be due to the spin-dipolar contribution, and indeed, solid-state DFT calculations confirm that the sign change is due to the contact SA.^64^

While the contact shift and SA are both due to the through-bond transfer of unpaired-electron-spin density to the nucleus and are thus short-range, the spin-dipolar contribution stems from the through-space coupling between the electronic and nuclear magnetic moments and therefore acts over longer distances exceeding 10 Å,^65^ allowing it to convey information about the mid- and long-range order of solids, typically obtained from XRD. In particular the PCS (isotropic component of term (iv) in Table I) has been employed as an additional restraint for the structure determination and refinement for crystalline samples of, e.g., rare-earth pyrochlores,^50,66^ or large biomolecules, such as metalloproteins.^67−69^ The PCS for the observed nucleus can be described as the sum of interactions with all paramagnetic centers within the same molecule (intramolecular PCS), and with any paramagnetic centers in adjacent molecules (intermolecular PCS).^67,69^ In an analogous way, information about the molecular and the crystal structure are both encoded in the PCS measured in solid-state materials.^68^ It is important to note that the precise determination of the PCS generally requires the availability of a diamagnetic analogue that does not undergo significant structural rearrangement upon incorporation of the paramagnetic metal ion and thus represents a suitable reference. While this might limit the applicability, purposeful paramagnetic doping on the other hand can provide additional insights: high dilution of the paramagnetic species may allow the separation of the intra- and intermolecular contributions to the PCS. Furthermore, the obtained structural information may be tuned by employing TM ions with smaller or larger magnetic susceptibility anisotropies. This also includes choosing slower or faster electronic relaxation, and with that a more or less pronounced PRE, as metalloproteins are one rare example where structural information has actually been extracted from comparing nuclear relaxation in diamagnetic and paramagnetic systems.^65,69,71−73^ This however typically involves a more complex analysis, since assumptions have to be made concerning the dominant nuclear relaxation mechanism and the relative time scales of the occurring dynamic processes, e.g., electronic relaxation and rotational diffusion. In addition, since transverse relaxation in solid-state materials is often driven by coherent contributions of all present nuclear spin interactions, the analysis is typically restricted to longitudinal relaxation times. Nonetheless, it is emphasized again that even without obtaining structural information, the PRE can still be very useful to significantly reduce acquisition times.^74,75^

Like the PCS, in solid materials, the spin-dipolar SA (term (iii) and anisotropic component of term (iv) in Table I) is the sum of contributions from adjacent paramagnetic centers, and the tensor describing the resulting interactions can deviate from axial symmetry. The resulting NMR powder lineshapes may be more complex, yet encode advanced geometric structure information that can be extracted upon careful analysis.^52,76,77^ An important example for obtaining the molecular and crystal structure for inorganic solids from both the spin-dipolar SA and the PCS is the investigation of the tris-dipicolinate lanthanide ion complexes shown in Figure 7a.^70^ As it was shown in Figure 3b and c, for isolated paramagnetic centers, the isosurfaces for the spin-dipolar SA and the PCS are relatively simple. However, for a lattice of paramagnetic centers, these isosurfaces become more complicated, but likewise richer in structural information. This is demonstrated in Figure 7b and c, respectively, where the corresponding interaction surfaces due to the sum of paramagnetic centers are superimposed on the lattice structure. In this fashion, the PCS, the spin-dipolar SA, and the associated asymmetry parameter give three parameters that can be obtained from calculations and experiment, and further used to optimize the respective atomic positions. The position-resolved ^1^H MAS NMR spectra of the lanthanide complex with Ln = Yb^3+^ and M = Cs^+^, used to determine the three parameters, are shown in Figure 7d. These spectra were extracted from a 2D ^1^H–^13^C transferred-echo double-resonance (TEDOR^23,78^) experiment. The comparison of the structures for this complex obtained from XRD (green) and from consulting the ssNMR parameters (colored) is given in Figure 7e. Of note, the overall average difference in atomic positions (measured in the root-mean-square deviation) for these two structures was found to be 0.22 Å, demonstrating the potential for molecular and crystal structure determination using paramagnetic ssNMR parameters.^70^

**Figure 7:**
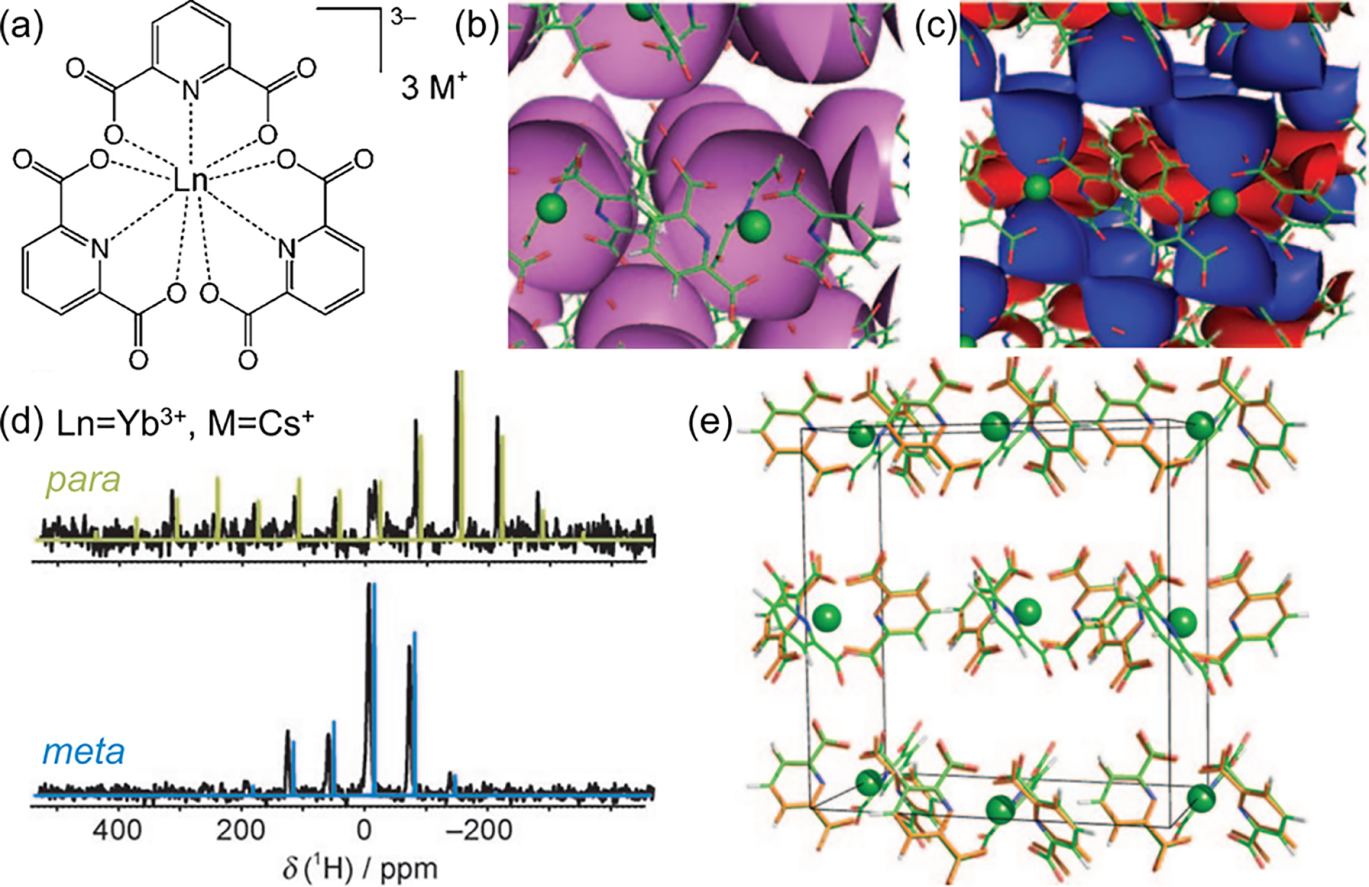
Determining the crystal structure using paramagnetic ssNMR. (a) Lanthanide compounds investigated in reference (70). The crystalline lattice for the compound given in (a) with Ln = Yb^3+^ is shown in parts (b) and (c). The lanthanide cation is indicated by the green sphere, where in the molecular structures oxygen is shown in red, carbon in green, nitrogen in blue, and hydrogen in gray. Isosurfaces for the spin-dipolar SA and the PCS (cf. Figure 3b and c) according to the crystalline lattice are, respectively, superimposed. (d) Position-resolved ^1^H MAS NMR spectra extracted from a 2D ^1^H–^13^C TEDOR NMR spectrum (33 kHz) of the complex shown in (a) for Ln = Yb^3+^ and M = Cs^+^. (e) Comparison of the structures for this complex known from XRD (orange) and obtained from ssNMR data (colored, as described above) for the unit cell. Adapted with permission from reference (70). Copyright 2009 WILEY-VCH Verlag GmbH & Co. KGaA, Weinheim.

The BMS contribution to the paramagnetic shift and SA for microcrystalline solids generally encodes information about the shape of the crystallites via **δ_in_^BMS^**, terms (a) and (b), and information about the size and packing of the crystallites contained in **δ_ext_^BMS^**, i.e., terms (c) and (d) in Table I. As described above, neither the shape and size of the crystallites nor the packing density is typically uniform within an investigated sample, such that both BMS contributions cause a distribution of paramagnetic shifts and SAs. The effect on the corresponding ssNMR spectrum is demonstrated in Figure 2b: under static conditions, a Gaussian line shape is expected, while MAS resolves the anisotropic line broadening into spinning-sideband manifolds. Note that the distribution of isotropic paramagnetic shifts (due to the ABMS terms (b) and (d) in Table I) is not removed by MAS, and manifests itself in the Gaussian line shape of the individual sidebands. This is likewise visible in Figure 2c, which shows the combined effect of orientational-dependent line broadening, stemming from local contribution **δ**^**S**^, and broadening due to BMS contribution **δ**^**BMS**^. Here, the distribution of paramagnetic SAs due to the BMS contribution causes a Gaussian smoothing of the sideband intensities (cf., Figure 2a and c). In particular for the static NMR powder signals, the sharp spectral singularities characterizing the respective local interaction are less well-accentuated (cf. Figure 2a). Therefore, the BMS contribution might be viewed as an additional source of inhomogeneous line broadening, that exacerbates the retrieval of the local structural information from the ssNMR spectrum. Accordingly, efforts have been made to remove BMS effects without likewise losing all local information, which might, e.g., be the case when applying ultrafast MAS to remove the anisotropic broadening. To this end, it has been suggested to embed the powdered sample of a paramagnetic system in a glassy matrix with equal magnetic susceptibility.^79,80^ In this approach, termed susceptibility matching, the effective magnetic boundary for the crystallite is the sample container and the demagnetizing anisotropies **Δ*****N***_***k*****≠0**_ effectively become zero for all crystallites, such that all terms due to adjacent crystallites are removed, i.e., terms (c) and (d) from Table I. It is, in particular, these anisotropic components and their distribution which are expected to dominate the BMS contribution to the paramagnetic SAs. A successful example for the application of susceptibility matching are the paramagnetic stannates Yb_2_Sn_2_O_7_ and Nd_2_Sn_2_O_7_, where impregnation with a solution of Er(NO_3_)_3_·5H_2_O in water has led to a significant reduction of BMS effects, and more clearly defined ^119^Sn MAS powder lineshapes could be obtained under slow MAS conditions (< 5 kHz).^81^ However, the practical difficulties of preparing a suitable susceptibility-matched matrix have limited this approach to further applications.

On the other hand, the BMS contribution does comprise morphological information about the crystallites, which, if measurable, might be able to fundamentally modify the range of insights obtained from ssNMR spectroscopy and yield information typically available from STEM. It is worth noting, however, that while the structural insights from ssNMR reflect the average morphological features of the whole microcrystalline solid, STEM data correspond to the local area chosen for examination. Exploring such new ssNMR territory has been rejuvenated by a series of recent studies concerning lithium-ion batteries: experimental results obtained from ^7^Li in situ NMR experiments on the electrode material LiMn_2_O_4_ have clearly demonstrated the extent to which the shape of the observed object and its relative orientation with respect to the external magnetic field contribute to both isotropic shifts and anisotropic broadenings, in particular for more extreme samples shapes as e.g., in battery pouch cells.^46^ Subsequent in-depth calculations confirmed that these BMS effects based on the dimensions, orientation and packing density of the present cells can be modeled numerically.^82^ The determination and, in particular, the interpretation of this information from ssNMR data is nevertheless an ongoing theoretical and experimental challenge. A potential strategy for obtaining insights about the average shape of the present crystallites constitutes in analyzing the ABMS contribution to the isotropic paramagnetic shift. Following the discussion above, this contribution stems from the isotropic components of the ABMS terms (b) and (d), or rather the expectation values of their respective underlying distributions (see Figure 4). As for term (d), this represents the packing and sizes of all present crystallites; central limit theorem predicts for the expectation value, and thus the net contribution to the paramagnetic shift to be zero.^13^ Assuming that all other contributions to the overall NMR shift for a sample are known, then the ABMS shift can be interpreted in terms of the expectation value for the distribution of the crystallite shapes. Such an approach has been applied to the well-characterized olivine-type lithium iron phosphates LiFePO_4_, as shown in Figure 8.^13^ While the data are not free of ambiguity, this demonstrates one of the first conceptual studies allowing a qualitative estimate for the average morphology of the crystallites based on BMS effects.

**Figure 8:**
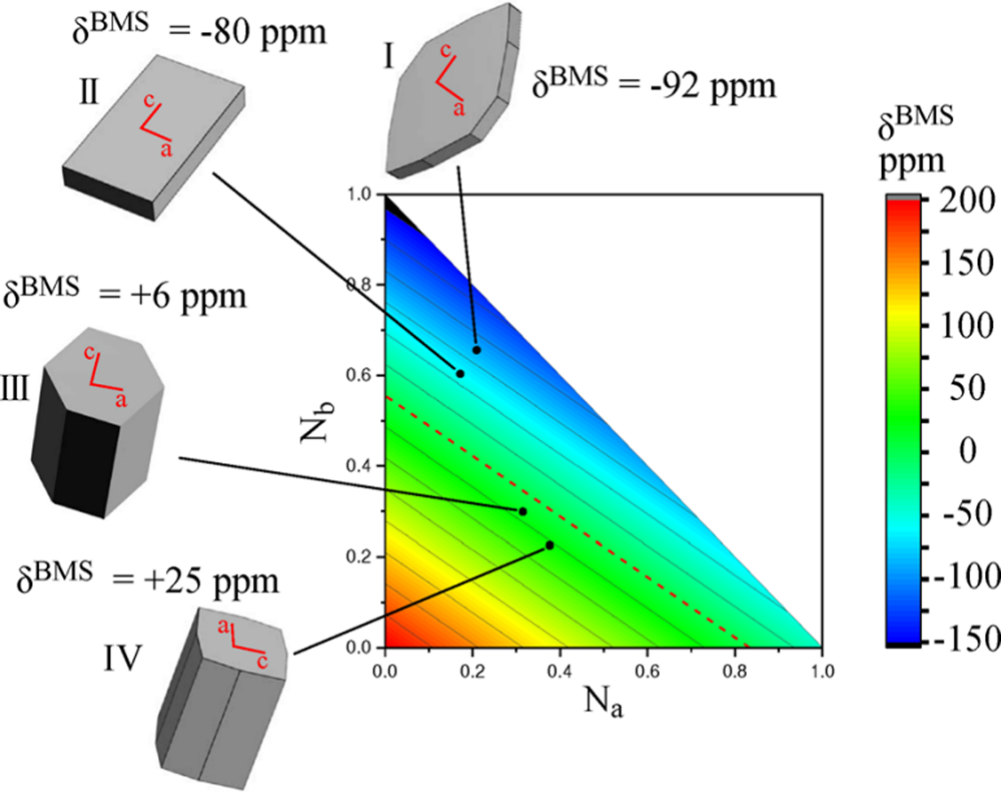
Analysis of the calculated BMS shift for LiFePO_4_ and its relationship to the crystal shape. Four representative morphologies (labeled I–IV) are represented as schematics in gray. For each crystallite, the (*a*, *c*) crystallographic axes are indicated in red on the face normal to the *b*-axis. The corresponding bulk magnetic susceptibility contribution of δ^BMS^ to the ^7^Li shift is also shown. Likewise indicated is the variation of the BMS shift as a function of the *N*_a_ (*x*-axis) and *N*_b_ (*y*-axis) principal components of the demagnetizing tensor ***N***. The variation with the third principal component *N*_c_ is not explicitly shown but can be inferred from *N*_c_ = 1 – (*N*_a_ + *N*_b_). The coloring in the plot represents the δ^BMS^ as determined by the relative (*N*_a_ vs *N*_b_) morphology of the LiFePO_4_ particle. The dotted red line indicates a BMS shift of 0 ppm. The points corresponding to the BMS shifts of the four crystal morphologies are indicated on the plot. Reproduced with permission from reference (13). Copyright (2019) American Chemical Society.

While the possibilities to obtain advanced structural insights from the BMS contribution have just started to be explored, the theoretical and experimental procedures for measuring and interpreting NMR shifts due to the local contributions are more sophisticated to date. Yet, the majority of ssNMR studies on paramagnetic materials have been focused on the paramagnetic shift, i.e., the contact shift and the PCS, while the anisotropic parts are, in general, less frequently considered. Since both parts contain congruent structural information (see Table I), the anisotropic ssNMR parameters can be used to reinforce the insights due the isotropic shifts. The focus on the isotropic components in recent years might in part be due to the fact that precisely measuring anisotropic broadenings in contrast to extracting isotropic shifts generally requires more advanced experimental techniques. Furthermore, extracting structural information often involves more complex theoretical considerations, e.g., the interpretation of the spin-dipolar SA that is due to several adjacent paramagnetic centers with different relative orientations and distances. Such more sophisticated analyses focusing on a single, dominant local interaction have been demonstrated to be very insightful in principle.^70^ However, to avoid any ambiguity in the analysis of experimental ssNMR data and thus structural misinterpretation, a unified theoretical approach is required that considers all local contributions simultaneously to both the paramagnetic shift and SA. While state-of-the-art computational methods applied to single molecules are capable of providing such high level of detail, merging these with solid-state calculations that include a solid grid and periodic boundary conditions constitutes a remaining challenge, even though recent theoretical advances are very promising.^63,83^ For the future, it is expected that elegant solutions potentially including machine-learning approaches will be developed that, in combination with high-resolution experimental data, ultimately may allow us to identify and precisely determine all contributions to the NMR shift due to paramagnetism. As we have laid out in this Perspective, the extent of the encoded structural information will result in moving beyond the current state, where paramagnetic ssNMR is mainly used to verify or refine structural information already known from alternative methods. This might be the full emancipation of paramagnetic ssNMR from the reliance of data from other techniques,^70,84^ since it has the potential to deliver comprehensive structural characterizations, including structural features ranging from particle morphologies to local atomic-structure subtleties.
